# Sequential infection with H1N1 and SARS-CoV-2 aggravated COVID-19 pathogenesis in a mammalian model, and co-vaccination as an effective method of prevention of COVID-19 and influenza

**DOI:** 10.1038/s41392-021-00618-z

**Published:** 2021-05-20

**Authors:** Linlin Bao, Wei Deng, Feifei Qi, Qi Lv, Zhiqi Song, Jiangning Liu, Hong Gao, Qiang Wei, Pin Yu, Yanfeng Xu, Yajin Qu, Fengdi Li, Jing Xue, Shuran Gong, Mingya Liu, Guanpeng Wang, Shunyi Wang, Binbin Zhao, Bin Cong, Chuan Qin

**Affiliations:** 1 Beijing Key Laboratory for Animal Models of Emerging and Remerging Infectious Diseases, NHC Key Laboratory of Human Disease Comparative Medicine, Institute of Laboratory Animal Science, Chinese Academy of Medical Sciences and Comparative Medicine Center, Peking Union Medical College, Beijing, China; 2 Hebei Medical University, Shijiazhuang, China

**Keywords:** Infectious diseases, Infectious diseases

## Abstract

Influenza A virus may circulate simultaneously with the SARS-CoV-2 virus, leading to more serious respiratory diseases during this winter. However, the influence of these viruses on disease outcome when both influenza A and SARS-CoV-2 are present in the host remains unclear. Using a mammalian model, sequential infection was performed in ferrets and in K18-*hACE2* mice, with SARS-CoV-2 infection following H1N1. We found that co-infection with H1N1 and SARS-CoV-2 extended the duration of clinical manifestation of COVID-19, and enhanced pulmonary damage, but reduced viral shedding of throat swabs and viral loads in the lungs of ferrets. Moreover, mortality was increased in sequentially infected mice compared with single-infection mice. Compared with single-vaccine inoculation, co-inoculation of PiCoVacc (a SARS-CoV-2 vaccine) and the flu vaccine showed no significant differences in neutralizing antibody titers or virus-specific immune responses. Combined immunization effectively protected K18-*hACE2* mice against both H1N1 and SARS-CoV-2 infection. Our findings indicated the development of systematic models of co-infection of H1N1 and SARS-CoV-2, which together notably enhanced pneumonia in ferrets and mice, as well as demonstrated that simultaneous vaccination against H1N1 and SARS-CoV-2 may be an effective prevention strategy for the coming winter.

## Introduction

Sequential viral infection is very common in clinical practice.^1,2^ Studies have reported that sequential infection of SARS-CoV-2 and influenza viruses is highly pandemic during the COVID-19 outbreak, and the co-infection rate is significantly enhanced compared to that during other periods.^1,3^ Sequential viral infection causes more serious disease and is difficult to identify, which causes significant concerns in clinical treatment.

Both influenza and SARS-CoV-2 viruses are respiratory tract viruses and enter the host through specific receptors, resulting in pneumonia in severe cases. Furthermore, the pathogenesis and receptors of these two viruses responsible for causing pneumonia are different^4^; the influenza infection affects the in upper respiratory tract while the SARS-CoV-2 infection primarily attacks the lower respiratory tract. Hence, there is no receptor competition, and sequential viral infection occurs without any difficulty. However, there is still no information about the consequences and mechanism of this sequential infection.^5–10^

Respiratory viruses are highly prevalent during the winter. Sequential infection of SARS-CoV-2 and influenza A, particularly the H1N1 virus, aggravates the patient’s condition, resulting in enormous difficulty in preventing and controlling the epidemic.^5–7,9^ This issue had attracted widespread attention. The key is to establish appropriate animal models for evaluating the possibility of sequential infection, investigate the consequences of sequential infection, and identify effective preventive measures.

In this study, ferrets and K18-*hACE2* mice, which are susceptible to both influenza and SARS-CoV-2,^11,12^ were used to establish animal models of sequential infection to investigate the consequences and pathogenesis of sequential infection of H1N1 and SARS-CoV-2. Further investigation and evaluation were conducted on K18-*hACE2* mice to determine the effectiveness of PiCoVacc (a SARS-CoV-2 vaccine),^13^ the flu vaccine, and a combined vaccine, and to validate the immune interference response in mice with combination immunization. A study on effective immunization strategy would provide foundational insights for the prevention and control of an epidemic.

## Results

### Initial infection with H1N1 aggravates pneumonia caused by SARS-CoV-2 in ferrets

**Fig. 1:**
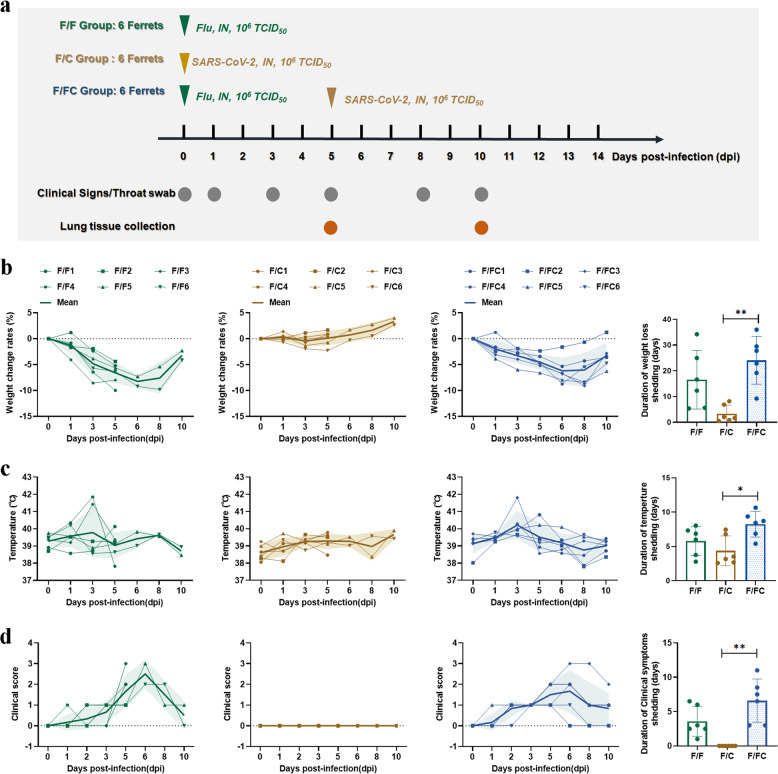
Clinical findings of co-infection with H1N1 and SARS-CoV-2 in ferrets. **a** Experimental design and sample collection. Eighteen ferrets were used in this study. Six ferrets were inoculated intranasally with H1N1 (1 × 10^6^ TCID_50_) and rechallenged intranasally with the same dose of SARS-CoV-2 (F/FC group, *n* = 6). The remaining ferrets were intranasally challenged with H1N1 (F/F group, *n* = 6) or SARS-CoV-2 (F/C group, *n* = 6) as control groups. Infected ferrets were observed for changes in body weight (**b**), body temperature (**c**), and clinical symptoms (**d**) at the indicated time points. Significant differences are indicated with asterisks (*P *<* 0.05, **P *<* 0.01*;* Student’s *t* test)

To investigate the pathogenesis of SARS-CoV-2 infection following the initial H1N1 infection, 18 adult ferrets were randomly divided into three groups. Six ferrets (F/FC1~6) were inoculated intranasally with H1N1 stock virus at 10^6^ tissue culture infectious dose (TCID_50_) and rechallenged with SARS-CoV-2 at the same dose at 5 days post-primary infection (dpi). Six ferrets were intranasally inoculated with SARS-CoV-2 (F/C1~6) at 10^6^ TCID_50_, and six ferrets were inoculated with H1N1 (F/F1~6) at 10^6^ TCID_50_ as single-infection control groups. The maximum weight loss (Fig. 1b) was 9.98% at 5 dpi, and the highest body temperature (Fig. 1c) was 41.85 °C at 3 dpi in the F/F group. The changes in body temperature and weight loss of F/C ferrets fluctuated within the normal range. However, in the F/FC group, body temperature fluctuated between 37.80 and 41.81 °C, and the maximum weight loss was 9.08% at 8 dpi (3 days post reinfection (dpr)), significantly different compared to the F/C group (Fig. 1b, c). The highest clinical score among F/F ferrets reached a peak value of 3, at 5–6 dpi, though the F/C ferrets did not exhibit obvious clinical symptoms (Fig. 1d). Notably, the highest clinical score in the F/FC group lasted for 3 days, between 6 and 8 dpi (1–3 dpr). The duration of clinical signs in the F/FC group was significantly increased compared with the F/C groups (Fig. 1d, right panel).

**Fig. 2:**
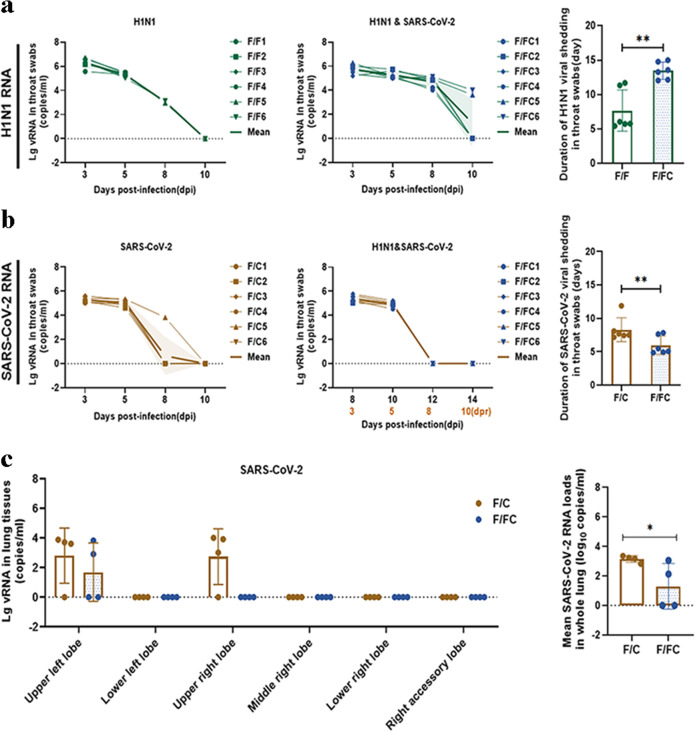
Viral characteristics following co-infection with H1N1 and SARS-CoV-2 in ferrets. **a** Viral RNA loads of H1N1 from throat swabs were detected by qRT–PCR (*n* = 6). **b** Viral RNA loads of SARS-CoV-2 from throat swabs were detected by qRT–PCR (*n* = 6). **c** Viral RNA of SARS-CoV-2 was measured in individual lung lobes of infected ferrets (*n* = 4). Significant differences are indicated with asterisks (*P < 0.05, **P < 0.01*;* Student’s *t* test)

**Fig. 3:**
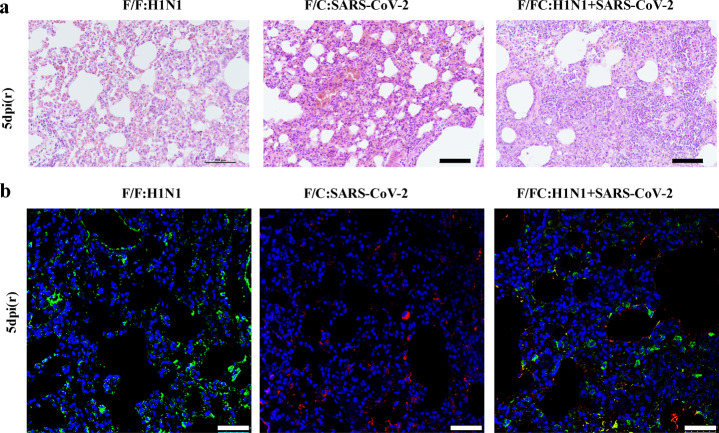
Histopathological features of co-infection with H1N1 and SARS-CoV-2 in ferrets. **a** Histopathological changes of F/F, F/C, and F/FC ferrets at 5 days post-virus challenge. **b** Immunofluorescence analysis of H1N1 or SARS-CoV-2 antigens in lung tissues from the F/F, F/C, and F/FC ferrets. Black bar = 100 μm, white bar = 50 μm

We next determined the viral RNA loads in the throat swabs during the 10 days after infection. H1N1 viral RNA in throat swabs reached the highest levels (10^6.71^ copies/ml) at 3 dpi. Viral shedding was detected at 8 dpi in F/F ferrets; however, viral shedding from throat swabs of two ferrets in the F/FC group lasted until 10 dpi (Fig. 2a). In addition, the SARS-CoV-2 virus shedding in the throat swabs of one F/C group ferret lasted until 8 dpi (viral loads:10^3.83^ copies/ml); however, there were no SARS-CoV-2 viral RNA loads detected at 8 dpr in the F/FC group, indicating that F/FC ferrets had reduced duration of viral shedding from throat swabs compared with the F/C group (Fig. 2b). At 5 dpi, four ferrets from these three groups were randomly euthanized for viral RNA detection and histopathological observation. Average SARS-CoV-2 RNA loads were observed in lung tissue in the F/C ferrets (10^3.63^ copies/ml in the upper left lobe and 10^3.69^ copies/ml in the upper right lobe, on average); however, the SARS-CoV-2 RNA load in the upper left lobe of the F/FC animals was 10^3.28^ copies/ml on average, and no viral RNA in the upper right lobe was detected from the F/FC animals (Fig. 2c, left panel); moreover, the average viral load in the entire lung in the F/FC animals was significantly decreased compared with that in the F/C group (Fig. 2c, right panel), suggesting that sequential H1N1 and SARS-CoV-2 infection inhibited the viral loads of SARS-CoV-2 in ferrets. Histopathological analyses revealed enlargement of the alveolar septum and inflammatory cell infiltration in lung tissues in both the F/F and F/C groups. Important differences included a greater broadening of the alveolar septum in the F/C group (Fig. 3a, middle panel), and more fulminant necrotizing and hemorrhagic pneumonia in the F/F group (Fig. 3a, left panel). However, lung tissues showed more severe necrotizing pneumonia, especially in the epithelial cells of the bronchi, greater inflammatory cell infiltration in the alveolar interstitium, and fibrin exudation in the alveolar cavities at 5 dpi in the F/FC ferrets, indicating that co-infection enhanced the development of pneumonia (Fig. 3a, right panel). Immunofluorescence staining for SARS-CoV-2 S protein and H1N1 HA protein demonstrated that a few alveolar epithelial cells were infected by the SARS-CoV-2 virus (Fig. 3b, middle panel) and some aggregated alveolar epithelial cells were infected by the H1N1 virus (Fig. 3b, left panel), respectively. Consistent with the pathological observation, at 5 dpi(r) in the F/FC ferrets, the expression of both viruses was observed in the alveolar epithelium (Fig. 3b, right panel).

### Co-infection with SARS-CoV-2 and H1N1 accelerated mortality in K18-*hACE2* mice

**Fig. 4:**
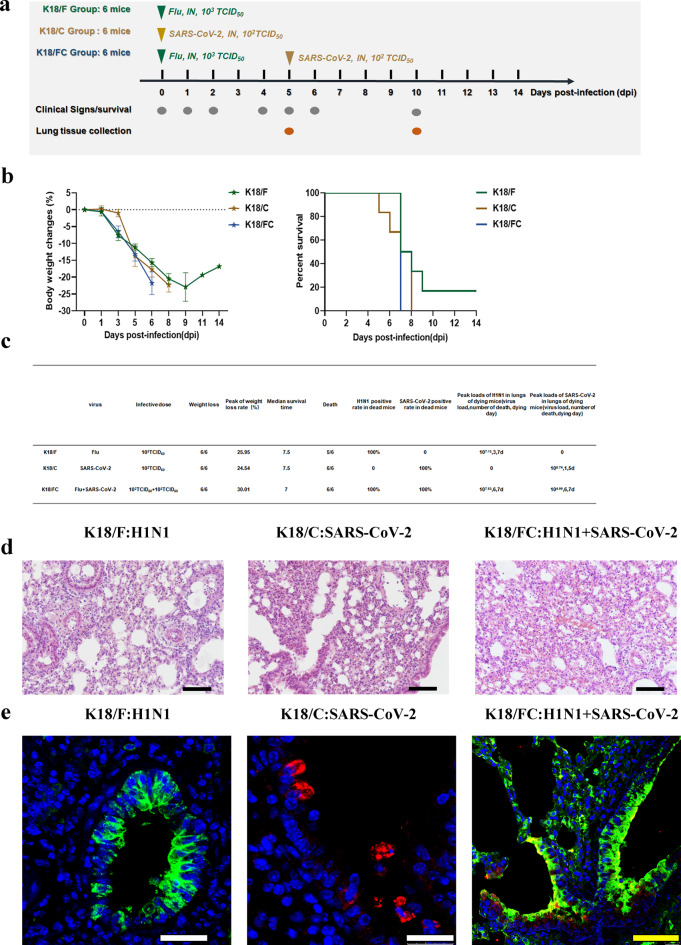
Co-infection of H1N1 and SARS-CoV-2 in K18-*hACE2* mice. **a** Experimental design and sample collection in K18-*hACE2* mice. **b** Changes in body weight and percent survival were recorded at the indicated time points in H1N1-infected K18-*hACE2* mice (K18/F), SARS-CoV-2-infected K18-*hACE2* mice (K18/C), and co-infected K18-*hACE2* mice (K18/FC) (*n* = 6). **c** Pathogenesis of H1N1 and SARS-CoV-2 in K18/F, K18/C, and K18/FC mice. **d** Histopathological changes in K18/F, K18/C, and K18/FC dying mice. **e** Immunofluorescence analysis of K18/F, K18/C, and K18/FC mice. Black bar = 100 μm, white bar = 25 μm, yellow bar = 75 μm

To further investigate the effect of H1N1 and SARS-CoV-2 co-infection on SARS-CoV-2-sensitive humans, K18-*hACE2* mice were used in this study.^12^ Study design and longitudinal sampling schedule are shown in Fig. 4a. After inoculation with the H1N1 virus (K18/F group) at 10^3^ TCID_50_, weight loss was observed from 1 to 9 dpi. One mouse recovered by ~9 dpi, the peak H1N1 RNA loads in the lungs of infected mice was 10^7.13^ copies/ml at 7 dpi (Fig. 4b, left panel; Fig. 4c). All mice inoculated with the SARS-CoV-2 virus (K18/C group) exhibited weight loss after 3 dpi, and none of these mice survived until the end of the observation period. The peak loads of the SARS-CoV-2 virus were 10^5.74^ copies/ml in the lungs of the infected mice (*n* = 1) (Fig. 4c). However, sequential infection with H1N1 followed by SARS-CoV-2 in mice (K18/FC group) quickened the rate of mortality, as all mice died within 7 dpi (2 days after co-infection). The average survival time of the co-infected mice (K18/FC) was 7 days, which was the shortest of the three groups (Fig. 4b, right panel; Fig. 4c). The peak viral loads of H1N1 and SARS-CoV-2 viruses in the lungs of co-infected mice from the K18/FC group were 10^7.53^ copies/ml and 10^4.99^ copies/ml, respectively. These results confirmed that co-infection accelerated death in K18-*hACE2* mice that were sensitive to SARS-CoV-2. Moreover, histopathological observation in the K18-*hACE2* mice was consistent with the results in ferrets. Lung tissues showed more severe inflammation in the alveolar interstitium, necrosis in the epithelial cells of the bronchi, and hemorrhage in the alveolar cavities in the K18/FC group, indicating that co-infection aggravated pneumonia in mice (Fig. 4d). Interestingly, compared with the distribution of SARS-CoV-2 in the alveolar epithelium (Fig. 4e, middle panel) in the K18/C group, the expression of H1N1 virus was abundant on the swollen and degenerated epithelial cells of the bronchi (Fig. 4e, left panel). After co-infection, the expression of both viruses was observed in pulmonary tissue (Fig. 4e, right panel).

### Immunological protection from H1N1 or SARS-CoV-2 infection in co-immunized K18-*hACE2* mice

We have confirmed that co-infection poses a major challenge to host health and that it is important to study the preventive effect of vaccines,^14^ especially the effects of co-immunization. To evaluate the effectiveness of combined immunization, K18-*hACE2* mice were randomly divided into three groups and immunized intraperitoneally with the PiCoVacc (SARS-CoV-2 vaccine, 3 μg/dose) and/or flu (3 μg/dose) vaccine (K18/CV, K18/FV, and K18/FCV groups). PBS-immunized K18-*hACE2* mice served as a control group (K18/CM, K18/FM).

**Fig. 5:**
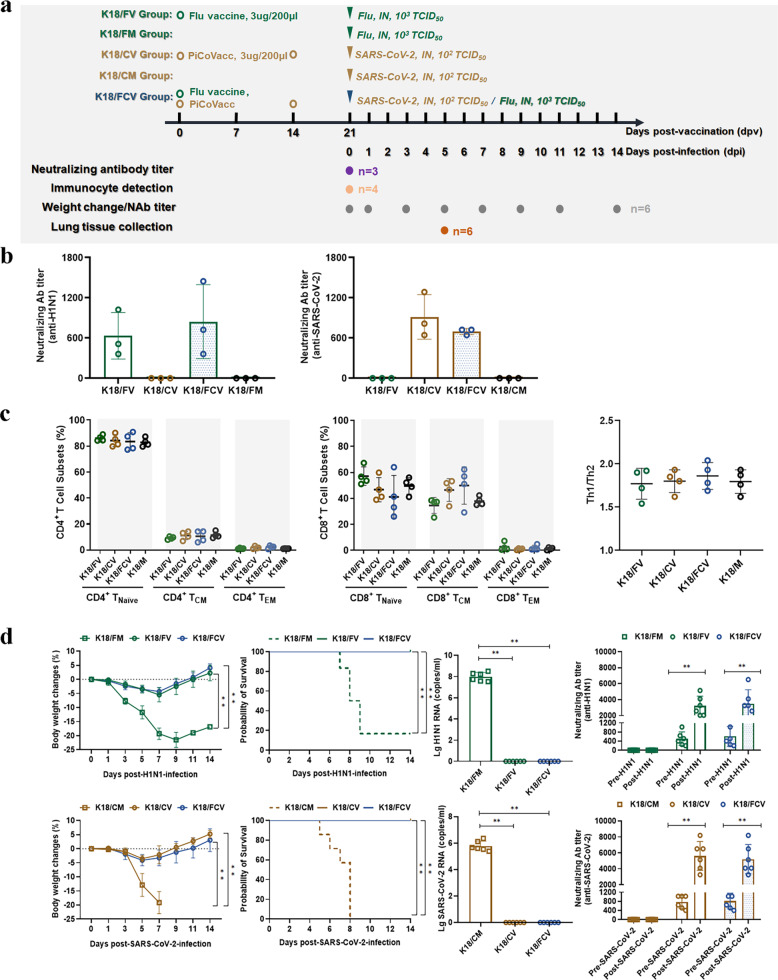
Comparison of cellular and humoral immunity among flu-vaccinated, SARS-CoV-2-vaccinated, or simultaneously immunized K18-*hACE2* mice. **a** Flu vaccine immunized mice (K18/FV), PiCoVacc (whole SARS-CoV-2-inactivated vaccine) immunized mice (K18/CV), and simultaneously immunized mice (K18/FCV). H1N1-infected K18-*hACE2* mice (K18/FM) and SARS-CoV-2-infected K18-*hACE2* mice (K18/CM) were used as infected controls. **b** Neutralizing antibody titers were measured in immunized K18-*hACE2* mice (*n* = 3). **c** Percentages of memory CD4^+^/CD8^+^ T-cell subsets from peripheral blood in co-infected mice (*n* = 4). **d** Changes in body weight and percent survival of mice immunized against SARS-CoV-2 or H1N1 infection at the indicated time points (*n* = 6, left panel). The viral RNA loads of H1N1 or SARS-CoV-2 were quantified at 5 dpi (*n* = 6, middle panel). The titers of neutralizing antibodies in K18-*hACE2* mice (with or without vaccine immunization) before and after H1N1 or SARS-CoV-2 infection (*n* = 6, right panel). Significant differences are indicated with asterisks (*P < 0.05; **P < 0.01; Student’s *t* test)

Mice from each group were euthanized at day 21 post immunization, and serum samples were tested for the presence of neutralizing antibodies (NAb). We found that NAb to SARS-CoV-2 was induced, ranging from 644 to 1287 in K18/CV mice. In the combined immunization group (K18/FCV), the NAb titers to SARS-CoV-2 ranged from 644 to 724 higher than the controls. Moreover, the NAb to H1N1 were raised equally in both individual immunization (K18/FV, 362–1024) and combined immunization groups (K18/FCV, 362–1448) (Fig. 5b). In addition, to investigate any immunopathology caused by SARS-CoV-2 or flu vaccines, T-cell responses were observed in immunized mice. Hematological and biochemical analysis of CD4^+^ or CD8^+^ T-lymphocyte subsets and Th1/Th2 ratio showed no notable changes in the K18/FCV group compared with individual immunization groups and nonvaccinated groups (Fig. 5c), suggesting no significant cell-mediated immunity against SARS-CoV-2 or H1N1 antigens in mice.

To further investigate the protective efficacy of combined immunization upon virus infection, the immunized mice were challenged with SARS-CoV-2 and H1N1, respectively. As shown in Fig. 5d, K18/CV, K18/FCV, and PBS-immunized K18/CM mice were inoculated intranasally with SARS-CoV-2 at a dosage of 10^2^ TCID_50_. Meantime, the K18/FV- and K18/FCV-immunized mice and K18/FM mice were infected with H1N1 at 10^3^ TCID_50_. Weight loss and mortality were monitored daily up to 14 days post inoculation. Mice of both the FV and FCV groups showed full protection against SARS-CoV-2 or H1N1 infection, as the mice both survived and maintained body weight (Fig. 5d, left panel). The NAb titers to SARS-CoV-2 from K18/CV (3259–8192) and K18/FCV (3259–8192) were remarkably increased by approximately fourfold compared to the levels before SARS-CoV-2 infection. Moreover, the NAb titers against H1N1 from K18/FV (2048–5149) and K18/FCV (1287–6517) were significantly enhanced after H1N1 infection, also approximately fourfold higher than before infection (Fig. 5d, right panel). In addition, no H1N1 or SARS-CoV-2 viral RNA was detected in the lungs of immunized mice after virus infection (Fig. 5d, middle panel), suggesting that combined immunization can offer full protective efficacy against the double insults of influenza and COVID-19. These results showed that combined immunization did neither interfere with the immune effect nor with the protective effect of the vaccine.

## Discussion

Upon sequential infection of H1N1 and SARS-CoV-2, pneumonia caused by COVID-19 was seen to be aggravated. The clinical manifestation of COVID-19 substantially varies largely due to natural host immunity, from asymptomatic or mild symptoms in immunocompetent hosts, to progressive pneumonia or even death in immunocompromised hosts.^15,16^ The initial infection of H1N1, which may reduce the immune competence of the hosts, can then aggravate experienced pneumonia by SARS-CoV-2 infection. Because the symptoms of SARS-CoV-2-infected ferrets are mild, a ferret is a suitable animal for the study of sequential infection. It is much easier to observe whether H1N1 infection will impair the immune surveillance for the entry of SARS-CoV-2, or sequentially aggravate the severity of SARS-CoV-2 infection in ferrets. We found that the mild pneumonia was obviously aggravated in sequential H1N1 and SARS-CoV-2-infected ferrets; however, the duration of viral shedding and the viral replication in lung tissue was also reduced in the ferrets. These results are consistent with other reports which stated that prior H1N1 infection followed by SARS-CoV-2 infection led to reduced SARS-CoV-2 pulmonary viral loads and enhanced lung damage in golden Syrian hamsters.^8^ The K18-*hACE2* mouse model, a susceptible model for SARS-CoV-2 infection, further confirmed the aggravation of pneumonia by H1N1-SARS-CoV-2 sequential infection. Secondary pneumonia following H1N1 and SARS-CoV-2 infection was robustly intensified. Using these two mammal models, our results demonstrated that host immunity-dependent COVID-19 symptoms could be aggravated by prior H1N1 infection.

Combined vaccination is commonly used for different viruses; however, the relevant data on co-vaccination against H1N1 and SARS-CoV-2 have not been reported. Using co-vaccination of H1N1 and SARS-CoV-2, neutralizing antibodies against H1N1 or SARS-CoV-2 were simultaneously induced and completely protected K18-*hACE2* mice against H1N1 and SARS-CoV-2. Compared to a single vaccination, there was no significant difference in the titers of neutralizing antibodies. All the vaccinated mice survived after H1N1 and SARS-CoV-2 infection (Fig. 5d); in addition, no antibody-dependent enhancement was observed. Further studies may explore the cross-reactivity of antibodies in combined vaccination.

In summary, vaccination is a priority to protect susceptible hosts from aggravation of COVID-19 symptoms. Co-vaccination against H1N1 and SARS-CoV-2 may be an effective system to protect against epidemic-related pneumonia in the coming winter.

## Materials and methods

### Ethics statement

All animal procedures were approved by the Institutional Animal Care and Use Committee of the Institute of Laboratory Animal Science, Peking Union Medical College (ILAS, PUMC) (No. BLL20010). All experiments were performed in an animal biosafety level 3 (ABSL3) facility with high-efficiency particulate air (HEPA)-filtered isolators.

### Virus

The SARS-CoV-2 virus designated as SARS-CoV-2/WH-09/human/2020/CHN (GenBank: MT093631.2) and the seasonal influenza A virus strain A/California/07/2009 (H1N1) were provided by ILAS, PUMC, China. To identify the stocks of the virus, the plaque purified viral isolate was amplified as described previously.^4^ Titers for SARS-CoV-2 and H1N1 were determined using a median TCID_50_ assay.

### Ferret experiments

Eighteen specific pathogen-free castrated adult ferrets (*Mustela putorius furo*) aged 6–12 months that were serologically negative for the currently circulating influenza viruses, MERS-CoV and SARS-CoV by hemagglutinin inhibition assay were used. Animals were randomly divided into three groups. Twelve ferrets (F/F1 to F/F6 and F/FC1 to F/FC6) were inoculated intranasally with H1N1 stock virus at 10^6^ TCID_50_. Following H1N1 infection, ferrets F/FC1 to F/FC6 were rechallenged with SARS-CoV-2 at 10^6^ TCID_50_. Six ferrets (F/C1 to F/C6) were inoculated intranasally with SARS-CoV-2 at 10^6^ TCID_50_, as a SARS-CoV-2 challenge control. All animals were observed for clinical signs and weighed daily as an indicator of disease. Nasal and throat swabs were collected on days 3, 5, 8, and 10 post infection. Four randomly selected animals were euthanized at 5 days post-H1N1 challenge and 5 days post-SARS-CoV-2 challenge to collect lung tissue for detecting viral loads and observing histopathological changes (*n* = 4). Study design and longitudinal sampling schedule are shown in Fig. 1a.

### Mice experiments

Eight–10-week-old female Tg (K18-*hACE2*) mice were provided by GemPharmatech Co., Ltd. Six transgenic mice (K18/FC group) were re-infected with SARS-CoV-2 at 1 × 10^2^ TCID_50_ at 5 days post-H1N1 infection at a dosage of 10^3^ TCID_50_. Six mice were challenged with H1N1 alone (K18/F group) at 10^3^ TCID_50_, and six mice were inoculated with SARS-CoV-2 alone (K18/C group) at 10^2^ TCID_50_. Infected animals were monitored and body weight, clinical symptoms, and responsiveness to external stimuli were recorded. Lung tissue was collected to screen virus loads and histopathological changes from the moribund mice infected with viruses.

### Vaccine immunogenicity analysis

Eight–10-week-old female Tg (K18-*hACE2*) mice were immunized intraperitoneally with the PiCoVacc (K18/CV, 3 μg/dose, SARS-CoV-2 vaccine, Sinovac Biotech Ltd), the flu vaccine (K18/FV, 3 μg/dose, Sinovac Biotech Ltd), or their combination (co-vaccination with both (K18/FCV)). PiCovacc-vaccinated animals were immunized two times (at day 0 and 14), and flu-vaccinated animals were immunized once (at day 0). The control group consisted of K18-*hACE2* mice immunized with PBS (K18/FM or K18/CM). To evaluate cellular immunity, four mice from each group were euthanized on day 21 post immunization. CD4^+^ or CD8^+^ T-lymphocyte subsets from blood were analyzed as follows: CD3^+^CD4^+^/CD8^+^CD62L^+^CCR7^+^CD44^−^ for naive T cells, CD3^+^CD4^+^/CD8^+^ CD62L^+^CCR7^+^CD44^+^ for central memory T cells, CD3^+^CD4^+^/CD8^+^CD62L^−^CCR7^−^CD44^+^ for effective memory T cells, CD3^+^CD4^+^IFNγ^+^ for Th1 cells, and CD3^+^CD4^+^IL-4^+^ for Th2 cells. Serum samples were analyzed for the presence of neutralizing antibodies by cytopathic effect (CPE) (*n* = 3). At day 21 post immunization, the K18/CV, K18/FCV, and K18/CM mice (*n* = 12) were inoculated intranasally with SARS-CoV-2 at a dosage of 10^2^ TCID_50_, and the K18/FV, K18/FCV, and K18/FM mice (*n* = 12) mice were infected with H1N1 at 10^3^ TCID_50_. Weight loss and mortality were monitored daily up to 14 days post inoculation (*n* = 6). Lung tissues were collected to quantitatively detect viral RNA loads at 5 days post infection (*n* = 6), and serum samples were collected to measure the titers of neutralizing antibodies to SARS-CoV-2 or H1N1 at 7 dpi.

### Quantification RT–PCR

The total RNA was extracted and reverse transcription was performed as described previously.^4^ Briefly, qRT–PCR was carried out using the following cycling protocol and primers: 50 °C for 2 min, then 95 °C for 2 min, followed by 40 cycles of 95 °C for 15 s and 60 °C for 30 s, and final incubations at 95 °C for 15 s, 60 °C for 1 min, and 95 °C for 45 s. The following primers were used to detect SARS-CoV-2 or H1N1: SARS-CoV-2: forward primer, 5′-TCGTTTCGGAAGAGACAGGT-3′; reverse primer, 5′-GCGCAGTAAGGATGGCTAGT-3′. H1N1: forward primer, 5′-GACCRATCCTGTCACCTCTGAC-3′, reverse primer, 5′-AGGGCATTYTGGACAAAKCGTCTA-3′.

### Statistical analysis

All data were analyzed with GraphPad Prism 8.0 software (GraphPad Software, Inc). Comparisons among groups were performed by the two-tailed unpaired Student’s *t* test. The level of statistical significance was determined as *P < 0.05, **P < 0.01.

## Data Availability

All data needed to evaluate the conclusions in the paper are present in the paper and/or the Supplementary Materials.
